# Dataset for characterization of dissolved organic matter extracted from organic wastes and their effects on the transport of titanium dioxide nanoparticles in acidic saturated porous media in the presence of monovalent electrolyte

**DOI:** 10.1016/j.dib.2019.105021

**Published:** 2019-12-20

**Authors:** Ruichang Zhang, Haibo Zhang, Chen Tu, Yongming Luo

**Affiliations:** a Chemical Engineering and Pharmaceutics School, Henan University of Science and Technology, Luoyang, 471023, PR China; b Key Laboratory of Soil Environment and Pollution Remediation, Institute of Soil Sciences, Chinese Academy of Sciences, Nanjing, 210008, PR China; c Key Laboratory of Coastal Environmental Processes and Ecological Remediation, Yantai Institute of Coastal Zone Research, Chinese Academy of Sciences, Yantai, 264003, PR China; d University of Chinese Academy Sciences, Beijing, PR China; e Key Laboratory of Soil Contamination Bioremediation of Zhejiang Province, Zhejiang A&F University, Hangzhou, 311300, PR China

**Keywords:** Transport, Titanium dioxide nanoparticles, Dissolved organic matter, Organic wastes, Monovalent electrolyte, DLVO theory, Zeta potential, Adsorption

## Abstract

This dataset is related to the research article in Chemosphere, entitled ‘The limited facilitating effect of dissolved organic matter extracted from organic wastes on the transport of titanium dioxide nanoparticles in acidic saturated porous media’ [1]. The data summarised the characterization of dissolved organic matter (DOM) extracted from organic wastes and their effects on the transport of titanium dioxide nanoparticles (TiO_2_ NPs) in acidic saturated porous media in the presence of monovalent electrolyte. Three types of dissolved organic matter were extracted from organic materials, including swine manure, sludge, and sediment, using deionized water, and were characterized with UV–Vis, FTIR and elementary analysis. The adsorption of DOM onto TiO_2_ NPs was evaluated in the presence of NaCl, and zeta potentials of TiO_2_ NPs were also determined. Breakthrough column experiments were conducted to quantify the effects of the extracted DOM on the transport behaviours of TiO_2_ NPs in acidic porous media compared with humic acid. Moreover, the interaction energy between nanoparticles and between nanoparticles and quartz media was calculated according to the classical DLVO theory. The dataset could be used as a reference for the evaluation and prediction of the environmental fate and subsequent risk of engineered nanomaterials.

**Table:** Specifications Table

Subject	Environmental chemistry
Specific subject area	Environmental behaviours and ecological impacts of engineered nanomaterials
Type of data	Tables and figures
How data were acquired	Elemental analysis (Vario Micro cube, Elementar, Germany) UV–Vis analysis (GENESYS 10S UV–Vis, Thermo Fisher, USA) FTIR analysis (Nicolet iS10, Thermo Scientific, USA) Total organic matter analysis (TOC-VCPN, Shimadzu, Japan) Zeta potential analysis (Zetasier Nano ZS90, Malvern Instruments, UK)
Data format	Raw and analyzed
Parameters for data collection	Titanium dioxide nanoparticles (TiO_2_ NPs) were purchased from Shanghai Aladdin Regent Co., Ltd. Humic acid (HA) was purchased from Sigma-Aldrich Chemical Co., Ltd. Quartz sand and other analytical grade chemicals were purchased from Sinopharm Chemical Regent Co., Ltd. Ultrapure Milli-Q water was used for the preparation of solutions and nanoparticles suspensions. Experiments were carried out at room temperature.
Description of data collection	Elementary composition, UV–Vis spectra and FTIR spectra of DOM and zeta potentials of TiO_2_ NPs were obtained from instrumental analysis. Interaction energy between nanoparticles and between nanoparticles and media was calculated according to the classical DLVO theory. DOM concentration before and after adsorption by TiO_2_ NPs was analyzed by TOC-VCPN. TiO_2_ NPs in the influent and effluent were quantified spectrophotometrically at a wavelength of 343 nm with the UV–Vis spectrometer.
Data source location	Henan University of Science and Technology, China
Data accessibility	With the article
Related research article	Ruichang Zhang, Haibo Zhang, Chen Tu, Yongming Luo. The limited facilitating effect of dissolved organic matter extracted from organic wastes on the transport of titanium dioxide nanoparticles in acidic saturated porous media. Chemosphere, 2019, 237: 124529 https://doi.org/10.1016/j.chemosphere.2019.124529 [1]

**Table:** 

**Value of the Data** • This data report the distinct facilitated effects of dissolved organic matter extracted organic wastes on the transport of TiO_2_ NPs in acidic porous media in the presence of monovalent electrolyte in comparison with HA. • This data are valuable to other scientists dedicating to investigating the environmental behaviours of engineered nanomaterials. • This data could be used as a reference for the evaluation and prediction of the environmental fate and subsequent risk of engineered nanomaterials. • Effects of DOM derived from natural organic materials on environmental behaviours of engineered nanomaterials can not be ignored in the future.

## 1 Data

**Fig. 1:**
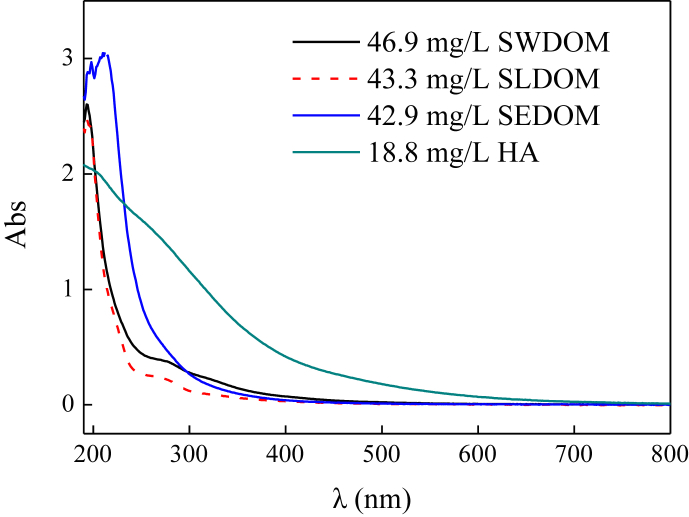
UV–Vis absorbance spectra of the extracted DOM and HA.

**Fig. 2:**
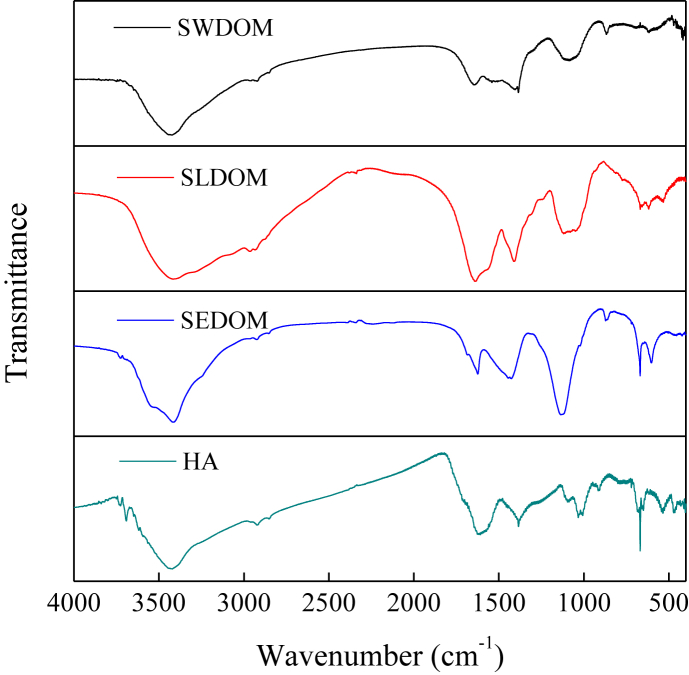
FTIR spectra of the extracted DOM and HA.

**Fig. 3:**
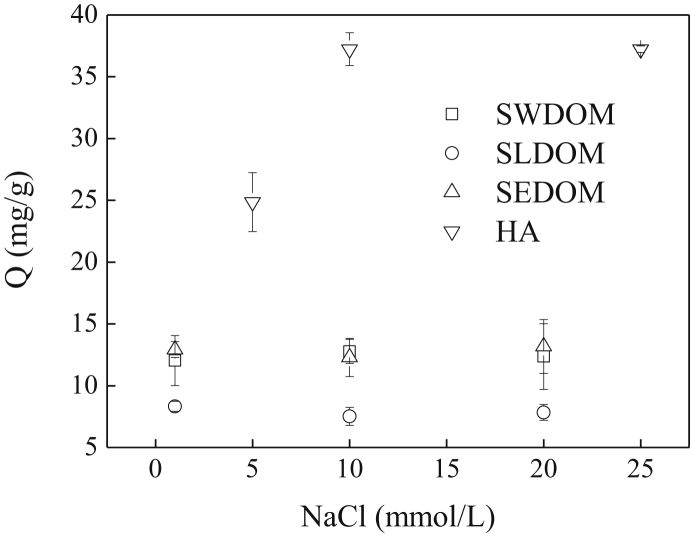
Adsorption of DOM onto TiO_2_ NPs in presence of NaCl.

**Fig. 4:**
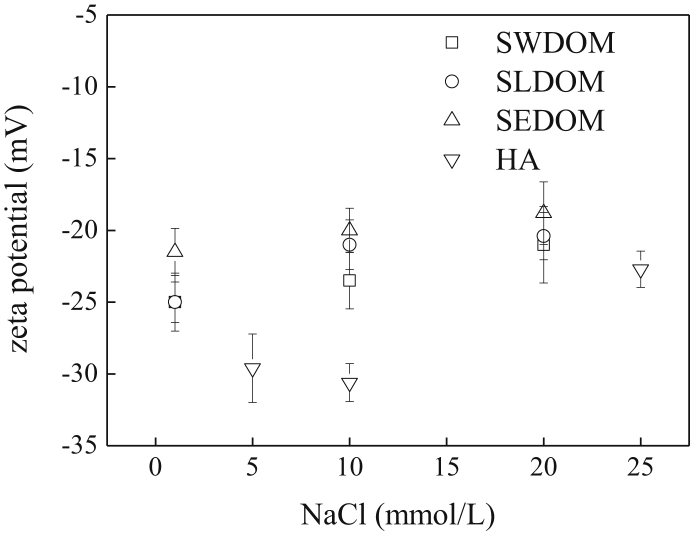
Zeta potential of TiO_2_ NPs in the presence of NaCl.

**Fig. 5:**
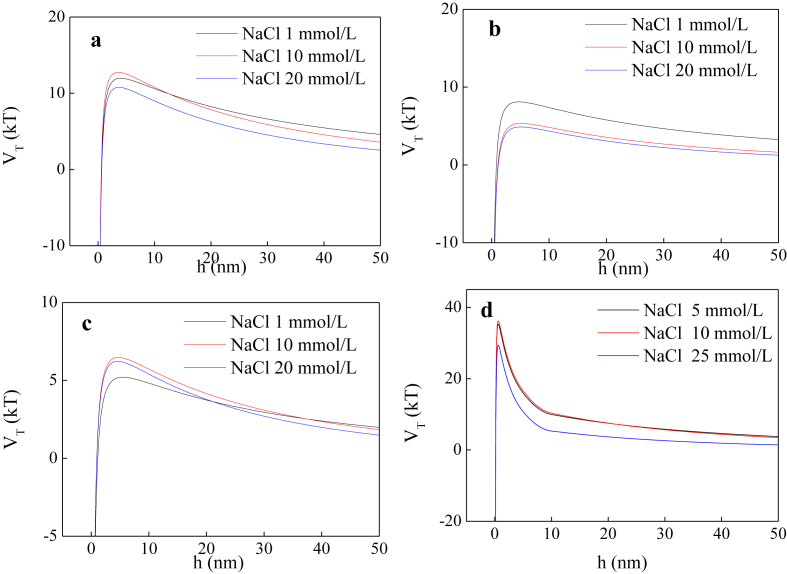
Calculated DLVO interaction energy between TiO_2_ NPs under varying NaCl concentrations in the presence of SWDOM (a), SLDOM (b), SEDOM (c) and HA (d).

**Fig. 6:**
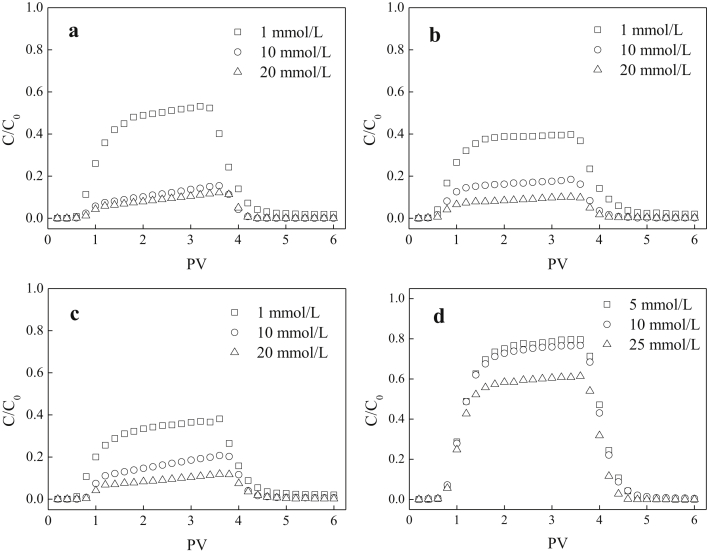
Breakthrough curves for TiO_2_ NPs at different NaCl concentrations in the presence of SWDOM (a), SLDOM (b), SEDOM (c), and HA (d).

**Fig. 7:**
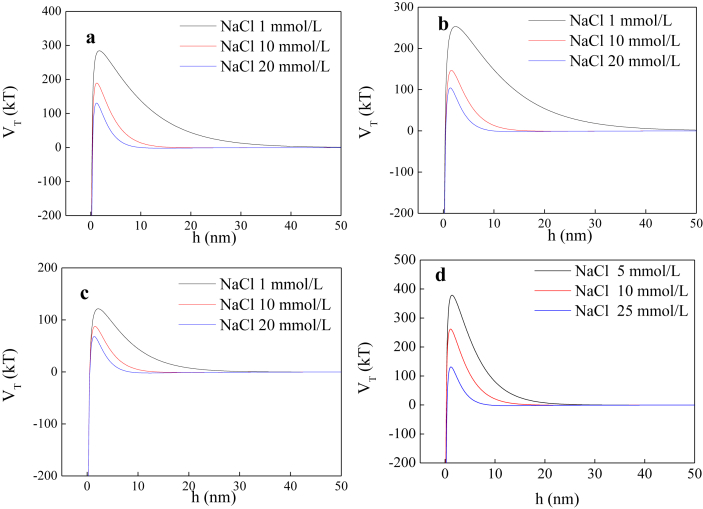
Calculated DLVO interaction energy between TiO_2_ NPs and quartz sand under varying NaCl concentrations in the presence of SWDOM (a), SLDOM (b), SEDOM (c) and HA (d).

The current dataset contains 2 tables and 7 figures. Elementary compositions (C, H, O, N, S and H/C) and ash contents of the extracted DOM and HA are given in Table 1. Fig. 1 shows the UV–Vis absorbance spectra of the extracted DOM and HA at wavelength of 190–800 nm. The tested concentrations were 46.9, 43.3, 42.9, and 18.8 mg/L for swine manure-derived DOM (SWDOM), sludge-derived DOM (SLDOM) and sediment-derived DOM (SEDOM) and HA respectively. All DOM concentrations in this study are reported as the dissolved TOC concentrations and not the total mass concentrations. FTIR spectra of the extracted DOM and HA at wavenumbers range of 400–4000 cm^−1^ and the assignment of absorption bands in FTIR spectra are shown in Fig. 2 and Table 2 respectively. Fig. 3 shows the adsorption of the DOM onto TiO_2_ NPs in the presence of 1, 10 and 20 mmol/L NaCl for the extracted DOM and 5, 10 and 25 mmol/L NaCl for HA. Fig. 4 shows the zeta potentials of TiO_2_ NPs in the presence of DOM and NaCl. Fig. 5 represents the calculated DLVO interaction energy between TiO_2_ NPs under varying NaCl concentrations in the presence of 8 mg/L SWDOM (a), SLDOM (b) and SEDOM (c) and 2 mg/L HA (d). Fig. 6 shows the breakthrough curves of TiO_2_ NPs in acidic porous media at different NaCl concentrations in the presence of SWDOM (a), SLDOM (b), SEDOM (c), and HA (d). Fig. 7 represents the calculated DLVO interaction energy between TiO_2_ NPs and quartz sand under varying NaCl concentrations in the presence of SWDOM (a), SLDOM (b), SEDOM (c) and HA (d). All raw data for the 7 figures are provided as Supplementary material.

**Table 1:** Elemental compositions of the extracted DOM and HA.

Sample	C	H	O	N	S	Ash	H/C
	(mass %)						
SWDOM	26.06	3.14	23.44	4.41	3.68	39.28	1.45
SLDOM	24.87	3.85	20.54	3.33	3.37	44.04	1.86
SEDOM	19.02	2.36	14.98	2.26	2.67	58.71	1.49
HA	40.41	3.67	48.71	3.03	1.51	2.67	1.09

**Table 2:** The assignment of absorption bands in FTIR spectra.

Wavenumber (cm^−1^)	Assignment
3500–3400	OH-stretching vibration of phenol, hydroxyl and carboxyl groups
3000–2800	Asymmetrical stretching vibration of C–H in aliphatics
1650–1600	Aromatic C=C stretching vibration and C=O stretching vibration of conjugated carbonyl groups
1450–1400	Stretching vibration of C–H in aliphatics, asymmetrical stretching vibration of carboxyl, and deformation vibration of C–OH
1260–1000	Aliphatic C–OH stretching vibration
870–640	Bending vibration of unsaturated band and benzene ring

## 2 Experimental design, materials, and methods

### 2.1 Materials

The nanoparticles, quartz sand and HA used in this study were identical to those used in our previous work [1,2]. TiO_2_ NPs were purchased from Shanghai Aladdin Reagent Co., Ltd. They were spherical with a nominal size of 30 ± 10 nm and a specific surface area of 80.8 m^2^/g. The crystalline composition was determined to be a pure anatase phase, and the point of zero charge was 6.2 in deionized water (18.2 MΩ cm). TiO_2_ NPs were used as received in all experiments. Stocking suspension of TiO_2_ NPs was prepared by adding 250 mg TiO_2_ NPs to 1.0 L deionized water. The suspension was sonicated for 30 min (500 W, 40 kHz) with vigorous stirring at room temperature (25 °C) and stored no longer than 2 d at 4 °C. Quartz sand (40–70 mesh) with an average diameter of approximately 350 μm was purchased from Sinopharm Chemical Reagent Co., Ltd. The sand was purified according to the method of Litton and Olson [3]. Surface impurities were removed by soaking in 12 mol/L HCl for 24 h followed by rinsing with deionized water until the pH of the rinse solution matched that of the deionized water. The sand was then baked in a furnace (FB1400, Thermo Scientific, USA) at 120 °C for 1 h and the at 800 °C for 5 h. HA was obtained from Sigma-Aldrich Chemical Co., Ltd. Stocking solution of HA was prepared at 1 g/L in deionized water with a small amount of NaOH as hydrotropic agent, and was stored no longer than 2 d at 4 °C. Other analytical grade chemicals used in this study were purchased from Sinopharm Chemical Regent Co., Ltd.

### 2.2 Extraction of DOM

DOM derived from three natural organic materials were extracted by deionized water using certain dry solid/water ratios of 1:20, 1:20 and 1:10 for swine manure, sludge and sediment respectively in a rotating shaker at 180 rpm at 25 °C for 12 h. The suspensions were centrifuged at 3500×*g* (3 K 15, Sigma Laborcentrifugen) for 30 min and then filtered through a 0.45 μm cellulous acetate filter membrane. The filtrates were referred to as swine manure-derived DOM (SWDOM), sludge-derived DOM (SLDOM) and sediment-derived DOM (SEDOM). The extracted DOM solutions were stored no longer than 2 d at 4 °C.

### 2.3 Preparation of TiO_2_ NPs suspensions

Immediately prior to each experiments, aqueous TiO_2_ NPs suspensions containing a final TiO_2_ NPs concentration of 50 mg/L were prepared by diluting the stocking suspension in 8 mg/L extracted DOM or 2 mg/L HA solution at the desired NaCl concentrations (1, 10 and 20 mmol/L for the extracted DOM batches and 5, 10 and 25 mmol/L for HA batch), and were adjusted to pH 4.0 with 0.1 mol/L HCl. The mixtures were sonicated for 4 min at 25 °C to obtain homogeneous suspensions. The zeta potentials of the TiO_2_ NPs suspensions were determined using a Zeta potential analysis (Zetasier Nano ZS90, Malvern Instruments, UK).

### 2.4 Adsorption of DOM onto TiO_2_ NPs

After 24 h, the suspended nanoparticles in TiO_2_ NPs suspension prepared above were pelleted by sequential centrifugation [4]. Briefly, 10 mL suspension was added to Teflon centrifuge tubes and centrifuged for 20 minutes at 9400×*g*. Then 8 mL of supernatant was carefully withdrawn from each tube and transferred into another clean centrifuge tube for centrifugation. This procedure was repeated 3 times until the TiO_2_ NPs were completely removed from the solution. The DOM concentration in the supernatant was measured as TOC on a TOC analyzer (TOC-VCPN, Shimadzu, Japan). The adsorbed DOM was then determined by the difference between the initial and final DOM concentrations in the aqueous phase. Control experiments with TiO_2_ NPs-free solutions showed no variations in DOM concentrations before and after the centrifugation processes in the range of DOM concentrations tested.

### 2.5 Column transport experiments

**Fig. 8:**
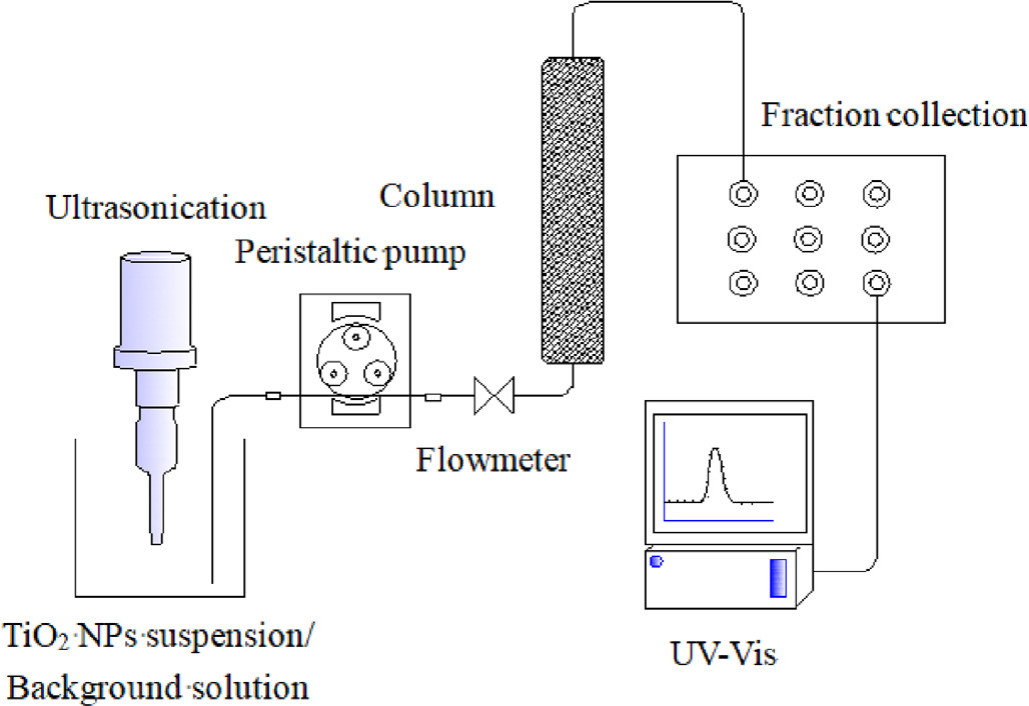
Schematic of transport experiments setup.

Schematic of transport experiments setup is shown in Fig. 8. Glass columns (2.5 cm in diameter and 10 cm in length) were packed uniformly with the purified quartz sand. The resulting porosity of the porous medium was gravimetrically measured to be 0.42–0.47. Once packed, the column was preconditioned with at least 10 pore volumes (PVs) of TiO_2_ NPs-free background solution with the desired DOM and NaCl concentration. Then, 3 PVs of TiO_2_ NPs suspensions (50 mg/L) with the same background chemistry were introduced into the column, followed by a nanoparticle-free background solution. The Darcy velocity was maintained at 0.35–0.38 cm/min for all experiments. The influent suspension was vigorously sonicated at room temperature to maintain consistent TiO_2_ NPs dispersion throughout the experiment. Column effluent was collected using a fraction collector (BS-110A, Huxi Analytical Instrument Factory Co., Ltd., China). The concentrations of TiO_2_ NPs in the influent (C_0_) and effluent (C) were determined spectrophotometrically at a wavelength of 343 nm. All transport experiments were conducted in duplicate with a deviation less than 3%; therefore, only one representative breakthrough curve for each experiment was shown in the results.

### 2.6 Derjaguin–Landau–Verwey–Overbeek (DLVO) theory

DLVO theory was applied to evaluate the role of electrostatic and van der Waals interactions on the interaction between the nanoparticles and the nanoparticle-quartz surfaces.

(1) $$\Phi_{Total}\left(h\right)=\Phi_{vdW}\left(h\right)+\Phi_{dl}\left(h\right)$$

DLVO interaction energies between TiO_2_ NPs were calculated assuming sphere-sphere geometry by utilizing the following equations [5]:

(2) $$\Phi_{vdW}\left(h\right)=-\frac{A_{101}a_{p}}{12h\left(1+14h/\lambda \right)}$$

(3) $$\Phi_{dl}\left(h\right)=2\pi \varepsilon_{0}\varepsilon_{r}a_{p}\psi_{p}^{2}\;\ln\left[1+\exp\left(-\kappa h\right)\right]$$

Interaction profiles for nanoparticles and quartz sand particles were developed assuming sphere-plate geometry and the following equations were used for calculation [5]:

(4) $$\Phi_{vdW}\left(h\right)=-\frac{A_{102}a_{p}}{6h\left(1+14h/\lambda \right)}$$

(5) $$\Phi_{dl}\left(h\right)=\pi \varepsilon_{0}\varepsilon_{r}a_{p}\left\{2\psi_{p}\psi_{c}\;\ln\left[\frac{1+\exp\left(-\kappa h\right)}{1-\exp\left(-\kappa h\right)}\right]+\left(\psi_{p}^{2}+\psi_{c}^{2}\right)\ln\left[1-\exp\left(-2\kappa h\right)\right]\right\}$$

In DLVO interaction energy profiles, positive interaction energy values represent repulsive condition whereas negative interaction energy values correspond to attraction.

When DLVO interaction energy between TiO_2_ NPs is calculated, *a*_*p*_ is the radius of the initial TiO_2_ NPs, 30 nm, and in the case of the energy between TiO_2_ NPs and quartz surface, the radius of an equivalent sphere for the nanoparticle aggregates which were measured by DLS has been used as the nanoparticle radius (*a*_*p*_). *h* denotes the (minimum) surface-to-surface separation distance between the spheres (for sphere–sphere geometry) or between a sphere and a plate (for sphere–plate geometry). A characteristic wavelength (*λ*) of 100 nm was assumed in the calculations. Permittivity of free space (*ε*_*0*_) and dielectric constant (*ε*_*r*_) of water are 8.854 × 10^−12^ C/V/m and 81.5 respectively, *κ* is the inverse Debye length (m^−1^) which was estimated for each electrolyte solution using eq. (6), and *ψ*_*p*_ and *ψ*_*c*_ are the surface potentials of TiO_2_ NPs and quartz collector (V), respectively. For the calculation of interaction profiles, zeta potentials of TiO_2_ NPs and quartz were measured under different chemical conditions and these values were used instead of surface potentials. The Hamaker constant for TiO_2_ NPs–water–TiO_2_ NPs interaction system (*A*_*101*_) used was 3.7 × 10^−20^ J [6] and for TiO_2_ NPs–water–quartz system (*A*_*102*_) 1.0 × 10^−20^ J was used [7].

(6) $$\kappa ={\left[\frac{10^{3}e^{2}N_{A}\left(2I\right)}{\varepsilon_{0}\varepsilon_{r}kT}\right]}^{1/2}$$

where *e* is the electron charge, 1.60 × 10^−19^ C, *N*_*A*_ is Avogadro's constant, 6.02 × 10^23^ mol^−1^, and *I* is the ionic strength of the solution.
